# Construction and internal validation of a risk prediction model for rapid bone loss during aromatase inhibitor therapy

**DOI:** 10.3389/fonc.2026.1854781

**Published:** 2026-08-26

**Authors:** Renda Jiang, Luning Wu, Liangying Zhao, Jiang Wang, Dalei Chen, Lingqiao Hu, Shiyu Wu, Chaoqun Chen, Guinv Hu

**Affiliations:** 1 Department of Breast Surgery, Dongyang Hospital Affiliated to Wenzhou Medical University, Dongyang, China; 2 Wenzhou Medical University, Wenzhou, China; 3 Sheng Jing Hospital Affiliated, China Medical University, Shenyang, China

**Keywords:** aromatase inhibitors, breast cancer, LASSO regression, nomogram, rapid bone loss

## Abstract

**Purpose:**

To establish and validate a risk prediction model for identifying hormone receptor-positive breast cancer patients at high risk of aromatase inhibitor-associated rapid bone loss.

**Methods:**

This single-center retrospective study enrolled 247 patients receiving aromatase inhibitor (AI) therapy between 2016 and 2025. Rapid bone loss, defined as an annual bone mineral density (BMD) loss rate greater than 4% at either the lumbar spine or total hip, was the primary outcome. Predictor selection was conducted using the least absolute shrinkage and selection operator (LASSO) regression. A multivariable logistic regression model was constructed to establish the predictive nomogram. Model performance was evaluated comprehensively using the area under the receiver operating characteristic curve (AUC) for discrimination, bootstrap calibration plots (1,000 resamples) with the Hosmer-Lemeshow (H–L) test for calibration, and decision curve analysis (DCA) for clinical utility.

**Results:**

Seven predictive indicators were incorporated into the final model: baseline hip BMD, body mass index (BMI), history of diabetes, high platelet-to-lymphocyte ratio (PLR), AI exposure before baseline DXA, red blood cell (RBC) count and follow-up interval. The nomogram shows acceptable discrimination after internal validation, with an optimism-corrected AUC of 0.709 (bootstrap, 1,000 resamples); the apparent AUC was 0.735 (95% CI: 0.672–0.798; sensitivity: 71.2%; specificity: 65.4%). Calibration was acceptable, with a bias-corrected calibration slope of 0.835 and a bias-corrected Brier score of 0.219; the apparent Hosmer–Lemeshow test was non-significant (P = 0.833). The DCA indicates potential clinical benefit within a wide range of threshold probabilities.

**Conclusion:**

This single-center study developed an exploratory prediction model that, after optimism correction, showed modest discrimination (corrected AUC 0.709) and may serve as a preliminary screening framework to identify postmenopausal patients at high risk of rapid bone loss related to early AI therapy. However, extensive prospective, multi-center external validation is strictly required before this model can be implemented to guide clinical decision-making.

## Introduction

Breast cancer is the most common malignant tumor among women worldwide, and its incidence rate has remained consistently high. Nearly 2.3 million new cases were reported globally in 2022, according to the World Health Organization (WHO). In China, the annual incidence approaches 400,000 (1). In all breast cancer subtypes, approximately 70% of patients are estrogen or progesterone receptor-positive (2). The progress of endocrine therapy has significantly improved clinical outcomes in postmenopausal patients with hormone receptor–positive (HR+) breast cancer, and AIs are now considered the standard of care for this population (3). However, the estrogen deprivation caused by AI treatment can disrupt bone remodeling and accelerate bone loss, which has become an important factor contributing to the decline in the quality of life of these patients. Current clinical guidelines mainly rely on static T-scores to guide intervention, whereas bone loss occurs most rapidly during the early phase of AI therapy (4).

Several approaches have been developed to identify patients at risk of AI-associated skeletal complications. The Fracture Risk Assessment Tool (FRAX), originally developed for the general population, has been evaluated in women initiating AI therapy and shown to stratify fracture risk reasonably well when BMD is incorporated into the calculation, although it was not specifically designed to capture the accelerated, therapy-induced component of bone loss (5). More recently, Chu et al. constructed a nomogram based on duration of breast cancer, hip fracture index, and prolactin level to predict the occurrence of AI-associated bone loss (AIBL) in 113 patients, reporting AUCs of 0.691–0.761 across logistic regression, LASSO, and XGBoost models (6). However, these and similar prior tools have generally defined the outcome as a static, binary state (e.g., FRAX-defined fracture probability or progression to osteopenia/osteoporosis by T-score) rather than the rate at which bone is being lost. Given that the most rapid phase of AI-induced bone loss occurs early in treatment—often before T-scores cross diagnostic thresholds—models based on static outcomes may fail to flag patients who are losing bone rapidly but have not yet reached osteopenic or osteoporotic status. Therefore, early identification of these “rapid losers” is useful for breast surgeons and oncologists in implementing timely, individualized preventative management.

## Study design and ethics

**Figure 1:**
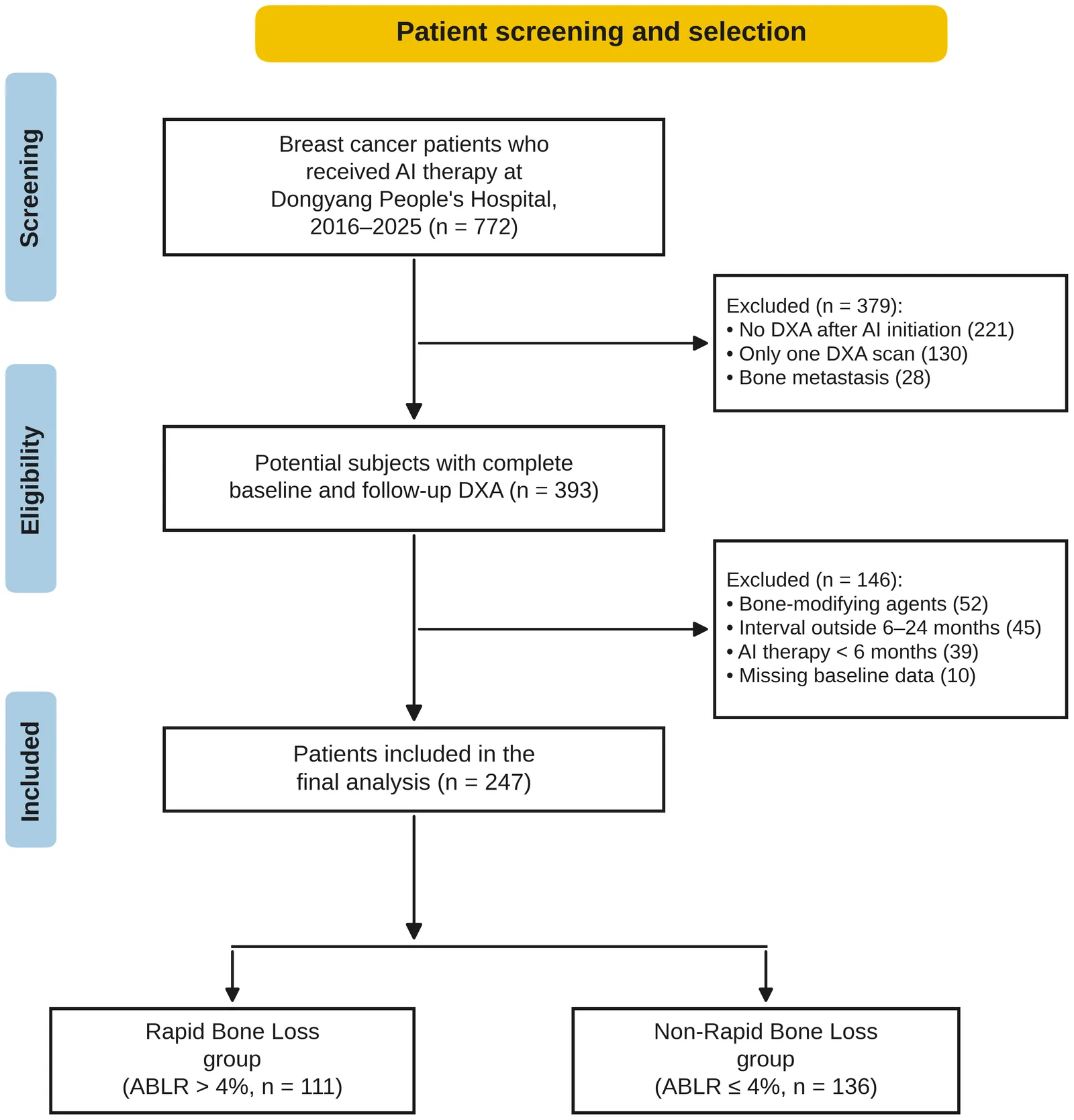
Schematic diagram of the research flowchart.

We conducted an observational, retrospective study at a single institution. Between January 2016 and December 2025, the medical records of individuals admitted with breast cancer to the Department of Breast Surgery at Dongyang People’s Hospital were systematically reviewed. The detailed research flowchart is depicted in **Figure 1**. This investigation received approval from the Medical Ethics Committee of Dongyang People’s Hospital (Approval 2026-YX-068) and complied with the Declaration of Helsinki. Since the study solely involved the retrospective analysis of de-identified clinical records, the requirement for written informed consent was waived.

## Participants

### Diagnostic criteria

The diagnosis of breast cancer and assessment of related biomarkers adhered to the guidelines of the Chinese Society of Clinical Oncology (CSCO) and the National Comprehensive Cancer Network (NCCN) (7). Diagnosis was confirmed via histopathological examination of core needle biopsy or surgical resection specimens, with HR+ status confirmed by postoperative immunohistochemistry. Following the 1994 WHO definitions, osteoporosis diagnoses were established through dual-energy X-ray absorptiometry (DXA) scans, with BMD classified by T-score thresholds as clinical osteoporosis (T-score ≤ −2.5), osteopenia (−2.5 < T-score < −1.0), or normal (T-score ≥ −1.0) (8).

### Inclusion criteria

Patients were included in the study if they met the following criteria:

1. Female patients diagnosed with breast cancer, with HR+ confirmed by postoperative immunohistochemistry;
2. Postmenopausal status (including natural menopause or menopause induced by ovarian function suppression) and aged ≥ 18 years;
3. Received adjuvant AI therapy (anastrozole, letrozole, or exemestane) for a total treatment duration of ≥ 6 months;
4. Completed two sequential DXA scans after the initiation of AI therapy, with an interval between scans ranging from 6 to 24 months (to calculate the annual bone loss rate (ABLR));
5. Availability of complete baseline clinical characteristics and laboratory data (with minor creatinine missingness permitted).

### Exclusion criteria

Patients were excluded based on the following criteria:

1. No DXA scan performed after AI initiation;
2. Only one DXA scan performed after AI initiation;
3. Evidence of bone metastasis at baseline or during the follow-up period;
4. Long-term use of bone-modifying agents before baseline or during follow-up (e.g., bisphosphonates, denosumab);
5. Inter-scan interval outside the specified range (6–24 months);
6. A duration of AI therapy of less than 6 months;
7. Missing key baseline clinical or laboratory data.

## Data collection and management

Relevant clinical parameters were comprehensively extracted, including treatment modalities, specific diagnostic features of the breast tumor, and patient baseline variables. Laboratory assessments included routine biochemical and hematological tests relevant to endocrine therapy. Fasting peripheral venous blood samples were obtained from all participants.

BMD at study entry was assessed using dual-energy X-ray absorptiometry at the lumbar vertebrae (L1–L4), femoral neck region, and total hip, using the same scanner (Prodigy, GE Healthcare) throughout the study period to ensure measurement consistency. The duration between the initial and the last DXA examinations was recorded and subsequently used to derive the ABLR. As routine DXA monitoring at our center is typically performed after AI initiation, the “baseline” DXA in this study refers to the first available scan after treatment initiation, not a pre-treatment measurement.

**Figure 2:**
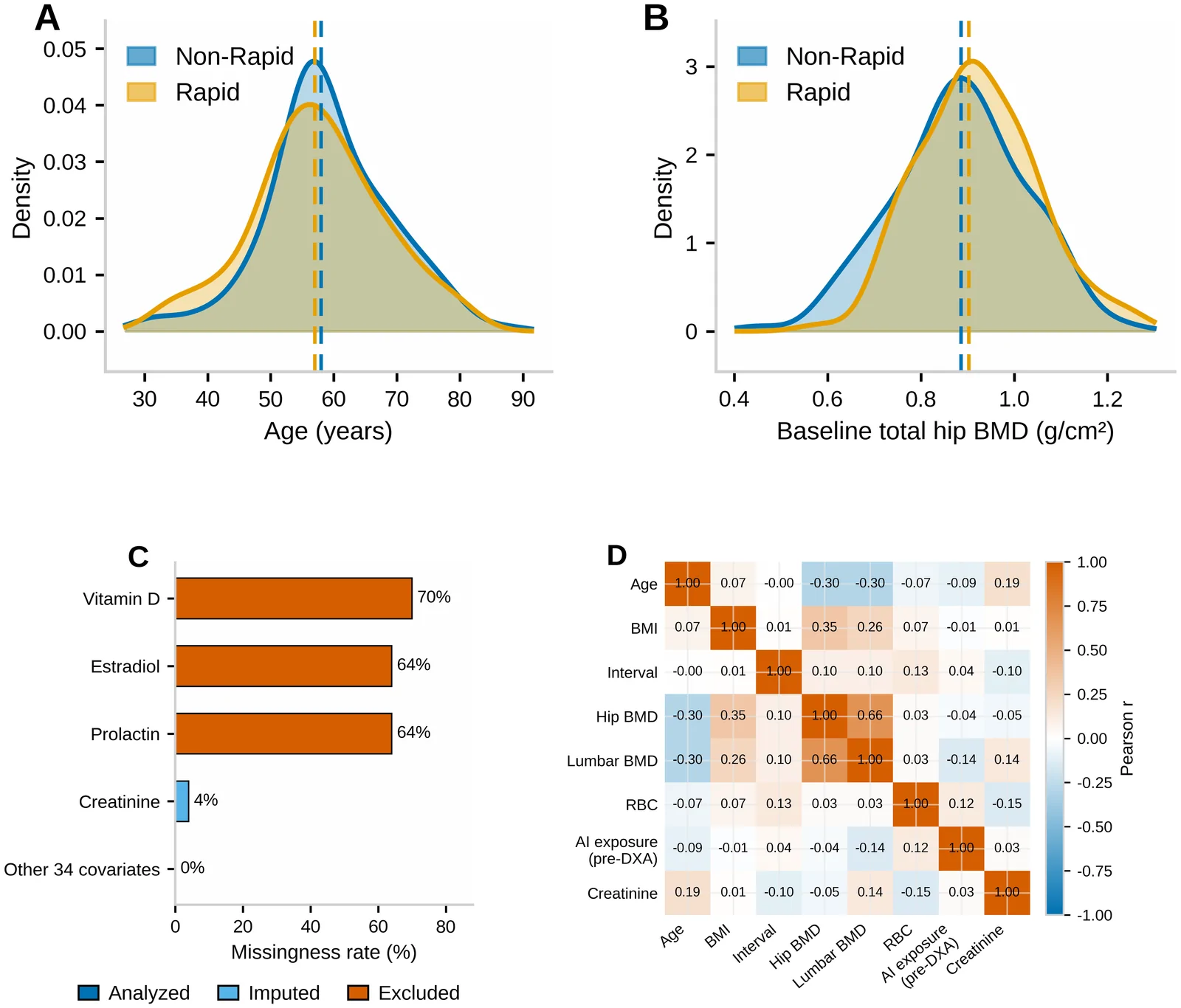
Baseline data exploration and quality assessment. **(A, B)** Apparent density distributions of baseline age and hip BMD. **(C)** Missing-data summary displaying missingness rates and handling strategies (imputation vs. exclusion). **(D)** Pearson correlation heatmap showing low pairwise correlation coefficients (r<0.40) among continuous candidate predictors.Abbreviations: BMD, bone mineral density; BMI, body mass index; PLR, platelet-to-lymphocyte ratio; RBC, red blood cell.

Data were retrospectively obtained from the electronic medical record system of Dongyang People’s Hospital. Extraction was performed independently by two investigators to enhance data reliability. All personally identifiable information was removed prior to analysis to ensure patient confidentiality. The dataset was then cleaned to correct logical inconsistencies and manage extreme outliers. Any discrepancies encountered during data collection were resolved through consultation with a senior reviewer. Missing data were handled according to each variable’s missingness rate. Serum creatinine (4.0% missing) was median-imputed to preserve statistical power and avoid selection bias. Biomarkers with severe missingness—vitamin D (70.0%), prolactin (64.0%), and estradiol (64.0%)—were excluded from modeling to avoid imputation bias. Definitions, coding, and missing rates for all variables are detailed in **Supplementary Table 1**, and the systematic missing-data summary is visualized in **Figure 2C**.

AI exposure before the baseline DXA scan (i.e., the interval from AI initiation to the first DXA examination) was recorded separately and analyzed as a candidate predictor, distinct from the total treatment duration used for eligibility.

## Definition of primary outcome and cutoff rationale

The main endpoint of the present study was the ABLR, calculated independently for the lumbar spine and total hip as:


$$\text{ABLR}(\%)=\frac{{\text{BMD}}_{\text{baseline}}-{\text{BMD}}_{\text{follow–up}}}{{\text{BMD}}_{\text{baseline}}\times \text{Interval}(\text{years})}\times 100\%$$

According to the derived ABLR values, participants were classified into two categories by applying a “worst-site” approach, whereby the skeletal site exhibiting the greatest rate of bone loss was used for grouping in order to enhance sensitivity of detection (the distribution of ABLR at the lumbar spine, total hip, and worst-site is illustrated in **Supplementary Figure 1**). Patients exhibiting an ABLR > 4% at either the lumbar spine or the total hip were defined as the “Rapid Bone Loss” group (positive outcome). Patients with an ABLR ≤ 4% at both sites were classified as the “Non-Rapid Bone Loss” group (negative outcome).

The 4% cutoff was selected based on clinical evidence and technical standards. In the pivotal ATAC trial, patients receiving anastrozole lost an average of 6.08% BMD at the lumbar spine over 5 years, with the most rapid loss occurring in the first 2 years (median change -4.1%) (9). We therefore defined rapid bone loss as an annual rate > 4% to identify patients whose loss trajectory exceeds this accelerated average. This threshold also exceeds the Least Significant Change (LSC, ~2–3%) defined by ISCD Official Positions (10), providing a conservative margin against measurement variability; the 2.5% threshold used in the sensitivity analyses below is a literature-based reference value, as no center-specific precision assessment was performed. To evaluate the robustness of our predictive model against different classification criteria, sensitivity analyses were further conducted using alternative ABLR thresholds: >2.5% (the literature-based threshold), >3.0%, and >5.0% (**Supplementary Figure 2**, **Table 5**).

## Statistical analysis

Continuous variables were presented as mean ± standard deviation (SD) or median (interquartile range, IQR), and compared using Student’s t-test or the Mann-Whitney U test, as appropriate. Categorical variables were expressed as frequencies (percentages) and analyzed using the Chi-square or Fisher’s exact test. Additionally, standardized mean differences (SMDs) were calculated to compare baseline characteristics between groups, with an SMD > 0.10 indicating a clinically meaningful baseline imbalance. Continuous hematologic indices that were dichotomized for analysis (PLR, neutrophil-to-lymphocyte ratio (NLR), and low-density lipoprotein cholesterol (LDL-C)) were categorized using data-driven, cohort-specific cutoff values derived from receiver operating characteristic (ROC) curve analysis (Youden index); these cutoffs were used for model development only and are not established clinical thresholds.

For model construction, variables with P < 0.1 in the univariate analysis alongside clinically relevant factors were entered into the LASSO regression. The clinical definitions, units, and coding systems applied to each predictor prior to LASSO are detailed in the clinical data dictionary (**Supplementary Table 9**). The optimal penalty parameter (λ) was determined via 10-fold cross-validation based on the 1-standard error (1-SE) criterion. Features with non-zero coefficients were subsequently incorporated into a multivariable logistic regression analysis to identify independent predictors. Prior to finalization, all core regression assumptions were systematically verified, including the absence of significant multicollinearity (assessed via Variance Inflation Factors [VIFs]), linearity of continuous predictors with the logit of the outcome (using the Box-Tidwell test), absence of influential outliers (using Cook’s distance), and the lack of statistically significant two-way interaction effects (tested via Likelihood Ratio Tests). These predictors were then used to construct the nomogram. The final model coefficients and point-scoring rules are summarized in **Supplementary Table 6**. Model performance was evaluated comprehensively: discrimination was quantified by the AUC; calibration was assessed using calibration plots with 1,000 bootstrap resamples and the H–L test; and clinical utility was estimated via the DCA. To benchmark the LASSO-logistic model against machine-learning algorithms, the cohort was first partitioned 7:3 into training and test sets. LASSO feature selection was performed using the training set only, and the resulting variables were used to train Random Forest and XGBoost classifiers; all models were evaluated once on the held-out test set to avoid data leakage. All statistical analyses were performed using R software (version 4.5.2). A two-sided P < 0.05 was considered statistically significant.

## Results

### Inclusion of patients

All eligible patients identified from the electronic medical record system completed baseline and follow-up BMD examinations, as well as tumor-related and blood index detection. A total of 247 subjects were included in the final analysis from January 2016 to December 2025. Due to the decade-long retrospective design, baseline data for the excluded patients (n=525) were not systematically extracted, precluding a formal statistical comparison. Nonetheless, a detailed breakdown of exclusion reasons (**Supplementary Table 2**) reveals that the majority of exclusions (66.8%) resulted from procedural or monitoring-related criteria (e.g., lack of a second DXA scan) rather than clinically meaningful differences in baseline characteristics. The median interval between the baseline and follow-up DXA scans was 1.1 years (IQR: 0.8–1.4). Based on the annual bone mineral density loss rate, 111 patients (44.9%) were classified into the Rapid Bone Loss group (> 4%), while the remaining 136 patients (55.1%) were classified into the Non-Rapid Bone Loss group. The baseline age and total hip BMD distributions of these two cohorts are visualized in **Figures 2A, B**, respectively. Additionally, a correlation heatmap confirmed the absence of significant pairwise multicollinearity among continuous candidate predictors (**Figure 2D**), and all Variance Inflation Factors (VIFs) in the final model were low (ranging from 1.05 to 1.15), supporting their suitability for subsequent multivariable modeling.

### Univariate analysis

The baseline demographic and clinical characteristics of the 247 patients are summarized in **Table 1**. In the univariate analysis, statistically significant differences were observed between the rapid bone loss and non-rapid bone loss groups in terms of history of diabetes (P = 0.003), BMI (P = 0.044), and follow-up interval (P = 0.036). With respect to skeletal parameters, the rapid bone loss group demonstrated significantly higher baseline BMD at both the lumbar spine (P = 0.050) and total hip (P = 0.023). In addition, hematologic indices revealed notable differences, with RBC count (P = 0.011) and hemoglobin (Hb) levels (P = 0.041) differing significantly between groups.

**Table 1:** Baseline demographic and clinical characteristics of the study population.

Variable	Non-rapid bone loss (N = 136)	Rapid bone loss (N = 111)	Total (N = 247)	SMD	P
Demographics and clinical characteristics					
Age, years	59.1 (9.76)	57.5 (10.3)	58.4 (10.0)	0.163	0.209
BMI, kg/m^2^	23.3 [21.3, 25.3]	24.2 [22.2, 26.3]	23.4 [21.5, 25.7]	0.248	0.044
Menopausal duration				0.038	0.122
≤5 years	47 (34.6%)	50 (45.0%)	97 (39.3%)		
>5 years	89 (65.4%)	61 (55.0%)	150 (60.7%)		
History of fractures				0.095	0.314
NO	117 (86.0%)	101 (91.0%)	218 (88.3%)		
YES	19 (14.0%)	10 (9.0%)	29 (11.7%)		
Hypertension				0.045	0.829
NO	89 (65.4%)	75 (67.6%)	164 (66.4%)		
YES	47 (34.6%)	36 (32.4%)	83 (33.6%)		
Diabetes				0.416	0.003
NO	117 (86.0%)	108 (97.3%)	225 (91.1%)		
YES	19 (14.0%)	3 (2.7%)	22 (8.9%)		
Bone mineral density					
Baseline Hip BMD, g/cm²	0.883 (0.139)	0.921 (0.125)	0.900 (0.134)	0.290	0.023
Baseline Lumbar BMD, g/cm²	0.994 (0.160)	1.04 (0.167)	1.01 (0.164)	0.253	0.050
Interval between DXA scans, years	1.13 [0.896, 1.50]	1.00 [0.750, 1.29]	1.08 [0.833, 1.38]	0.320	0.036
Tumor and treatment characteristics					
Clinical Stage				0.172	0.226
Stage I	57 (41.9%)	56 (50.5%)	113 (45.7%)		
Stage II-IV	79 (58.1%)	55 (49.5%)	134 (54.3%)		
T Staging				0.046	0.817
Stage I	79 (58.1%)	67 (60.4%)	146 (59.1%)		
Stage II-IV	57 (41.9%)	44 (39.6%)	101 (40.9%)		
Type of AI therapy				0.019	0.801
Letrozole	80 (58.8%)	65 (58.6%)	145 (58.7%)		
Anastrozole	50 (36.8%)	43 (38.7%)	93 (37.7%)		
Exemestane	6 (4.4%)	3 (2.7%)	9 (3.6%)		
Ca/VitD supplementation				0.126	0.419
NO	110 (80.9%)	95 (85.6%)	205 (83.0%)		
YES	26 (19.1%)	16 (14.4%)	42 (17.0%)		
AI exposure before baseline DXA, months	4.00 [0, 12.3]	3.00 [0, 10.0]	4.00 [0, 10.0]	0.322	0.338
Radiotherapy				0.012	1.000
NO	58 (42.6%)	48 (43.2%)	106 (42.9%)		
YES	78 (57.4%)	63 (56.8%)	141 (57.1%)		
Chemotherapy				0.018	0.993
NO	60 (44.1%)	48 (43.2%)	108 (43.7%)		
YES	76 (55.9%)	63 (56.8%)	139 (56.3%)		
Targeted Therapy				0.017	1.000
NO	116 (85.3%)	94 (84.7%)	210 (85.0%)		
YES	20 (14.7%)	17 (15.3%)	37 (15.0%)		
Glucocorticoids				0.018	1.000
NO	135 (99.3%)	110 (99.1%)	245(99.2%)		
YES	1 (0.7%)	1 (0.9%)	2(0.8%)		
Laboratory findings					
LDL-C status				0.180	0.203
Optimal (< 2.8 mmol/L)	61 (44.9%)	40 (36.0%)	101 (40.9%)		
Elevated (≥2.8 mmol/L)	75 (55.1%)	71 (64.0%)	146 (59.1%)		
Neutrophil-to-lymphocyte ratio (NLR)-status				0.037	0.879
Low (< 2.4)	92 (67.6%)	77 (69.4%)	169 (68.4%)		
High (≥ 2.4)	44 (32.4%)	34 (30.6%)	78 (31.6%)		
Platelet-to-lymphocyte ratio (PLR)	147.95 [108.35, 181.91]	139.60 [104.80, 201.39]	140.79 [107.06, 186.41]	0.197	0.822
PLR-status				0.244	0.080
Low (< 214)	117 (86.0%)	85 (76.6%)	202 (81.8%)		
High (≥214)	19 (14.0%)	26 (23.4%)	45 (18.2%)		
Red blood cell count, ×10^12^/L	4.28 [3.98, 4.47]	4.39 [4.13, 4.63]	4.32 [4.04, 4.55]	0.280	0.011
Alkaline phosphatase, U/L	89.0 [73.0, 101]	82.0 [68.0, 98.0]	85.0 [70.5, 101]	0.175	0.059
Creatinine, μmol/L	52.0 [47.8, 60.0]	52.0 [47.5, 58.0]	52.0 [47.5, 59.0]	0.097	0.594
Calcium, mmol/L	2.32 [2.25, 2.37]	2.33 [2.27, 2.41]	2.32 [2.26, 2.38]	0.186	0.128
Phosphorus, mmol/L	1.21 [1.11, 1.28]	1.21 [1.13, 1.30]	1.21 [1.12, 1.29]	0.087	0.496
Total bilirubin, μmol/L	11.0 [8.80, 14.0]	11.4 [9.35, 14.1]	11.3 [9.20, 14.0]	0.053	0.473
Total protein, g/L	72.5 [69.1, 75.8]	74.0 [70.1, 76.7]	72.9 [69.6, 76.1]	0.127	0.118
Alanine aminotransferase, U/L	18.0 [15.0, 29.3]	20.0 [15.5, 29.0]	19.0 [15.0, 29.0]	0.099	0.142
Aspartate aminotransferase, U/L	22.0 [18.8, 26.0]	23.0 [19.0, 28.0]	23.0 [19.0, 27.0]	0.134	0.234
Calcium phosphorus ratio	1.93 [1.80, 2.04]	1.94 [1.80, 2.09]	1.94 [1.80, 2.06]	0.048	0.886
Calcium phosphorus product	2.78 [2.53, 3.00]	2.83 [2.57, 3.06]	2.80 [2.54, 3.05]	0.151	0.253
White blood cell count, ×10^9^/L	4.96 [4.23, 5.84]	5.06 [4.13, 6.17]	5.04 [4.21, 5.92]	0.104	0.350
Neutrophil count, ×10^9^/L	2.78 [2.41, 3.39]	2.98 [2.24, 3.66]	2.85 [2.35, 3.53]	0.076	0.421
Lymphocyte count, ×10^9^/L	1.50 [1.18, 1.92]	1.59 [1.17, 2.01]	1.52 [1.18, 1.96]	0.007	0.775
Hemoglobin, g/L	129 [123, 135]	132 [125, 139]	131 [123, 137]	0.181	0.041
Platelet count, ×10^9^/L	219 (62.4)	224 (56.0)	221 (59.6)	0.089	0.487

SMD, Standardized Mean Difference; BMI, body mass index; BMD, bone mineral density; DXA, dual-energy x-ray absorptiometry; AI, aromatase inhibitor; LDL-C, low-density lipoprotein cholesterol; Cutoff values for PLR (214), NLR (2.4) and LDL-C (2.8) were determined by ROC curve analysis using the Youden index on the present dataset; they are exploratory, cohort-specific values rather than established clinical thresholds.

Continuous variables are presented as mean (SD) in parentheses, or median [IQR] in square brackets, Categorical variables are expressed as n (%).

In contrast, no statistically meaningful differences were observed for other variables, including age, duration of menopause, prior fracture history, tumor subtype, clinical stage, type of AI, continuous PLR (P = 0.822), and elevated binary PLR (P = 0.080) (all P > 0.05). Variables exhibiting intergroup differences were subsequently considered as candidate predictors for further feature selection procedures.

### Feature selection via LASSO regression

**Figure 3:**
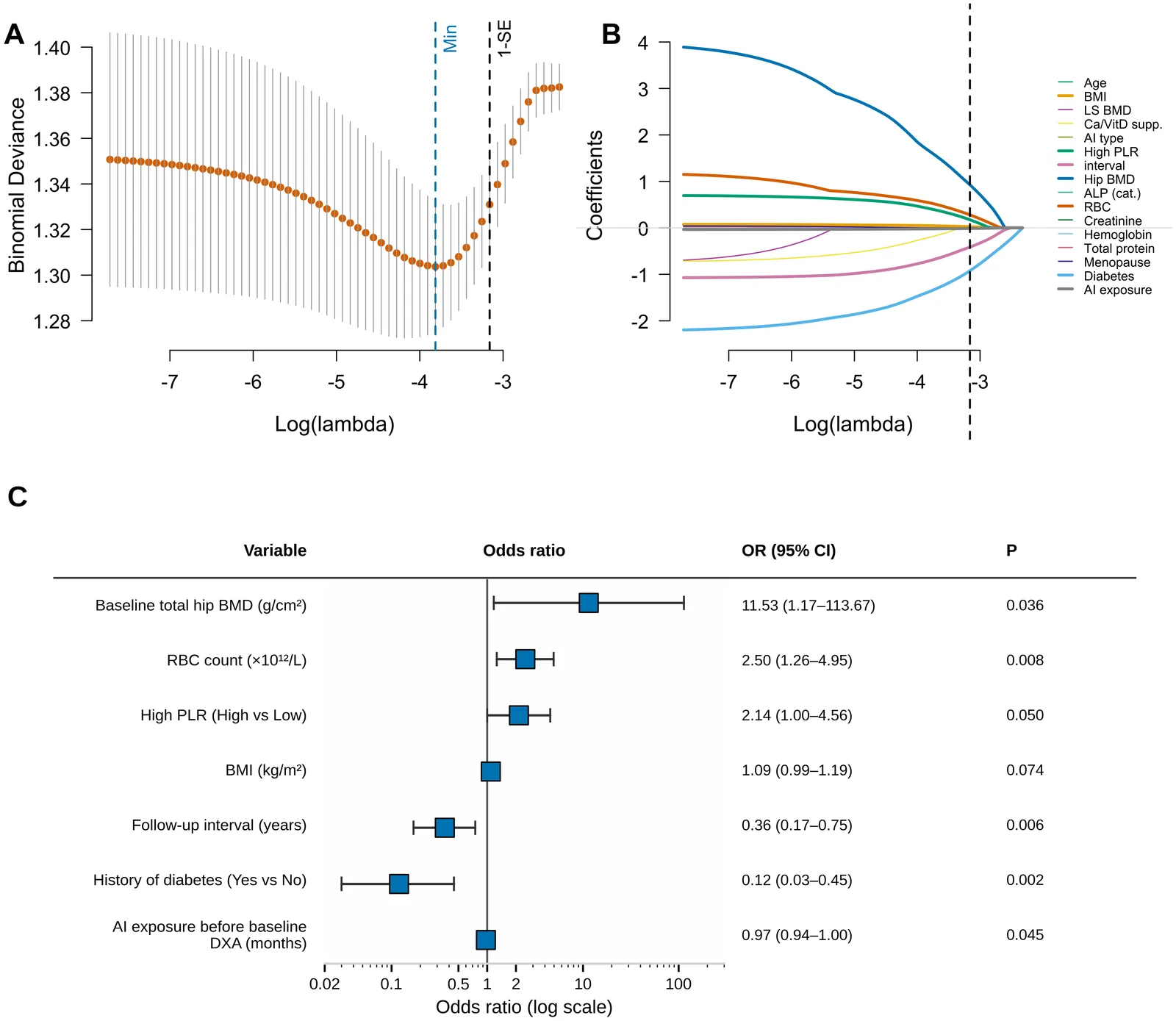
Variable selection and prediction model construction. **(A)** Ten-fold cross-validation based on binomial deviance, displaying the optimal penalty parameters (λmin=0.015, λ1se=0.041). **(B)** LASSO coefficient shrinkage trajectories plotted against the log(λ) sequence. **(C)** Forest plot of the final multivariable logistic regression model displaying Wald-test-derived Odds Ratios (ORs) and 95% confidence intervals (CIs). Abbreviations: LASSO, least absolute shrinkage and selection operator; OR, odds ratio; CI, confidence interval.

To mitigate the risk of overfitting and account for potential multicollinearity among candidate predictors, LASSO regression was applied for variable selection. A total of 16 variables—either demonstrating a P value < 0.1 in univariate analyses or deemed clinically relevant—were included in the model. The optimal tuning parameter (λ) was determined via ten-fold cross-validation. As illustrated in **Figure 3A**, the optimal λ was chosen according to the one-standard error (1-SE) rule. **Figure 3B** depicts the trajectories of regression coefficients across the log(λ) sequence. Ultimately, seven variables with non-zero coefficients were retained as potential predictors of rapid bone loss, including BMI, diabetes history, baseline total hip BMD, elevated PLR, AI exposure before baseline DXA, RBC count, and follow-up interval.

### Construction of the prediction model

The seven predictors selected via LASSO were subsequently entered into a multivariable logistic regression model to establish the predictive framework. The final model coefficients and selected variables are visually summarized in the clinical forest plot (**Figure 3C**) and detailed in **Table 2**. Variables identified as independent risk factors for rapid bone loss included elevated PLR (OR = 2.14, 95% CI: 1.00–4.56, P = 0.050), higher baseline total hip BMD (OR = 11.53, 95% CI: 1.17–113.67, P = 0.036), and increased RBC count (OR = 2.50, 95% CI: 1.26-4.95, P = 0.008). Conversely, history of diabetes (OR = 0.12, 95% CI: 0.03–0.45, P = 0.002), longer follow-up interval (OR = 0.36, 95% CI: 0.17–0.75, P = 0.006), and longer AI exposure before baseline DXA (OR = 0.97, 95% CI: 0.94–1.00, P = 0.045) were identified as significant protective factors (OR < 1). Although BMI (P = 0.074) demonstrated only marginal statistical significance in the multivariable model, it was retained in the final model based on its selection by LASSO, in order to preserve model stability and improve clinical interpretability. Additionally, all possible two-way interaction effects among the seven predictors were tested and found to be non-significant (all P>0.10). To maintain model parsimony, prevent overfitting, and ensure the intuitive legibility of the nomogram, no interaction terms were incorporated into the final model.

**Table 2:** Multivariable logistic regression analysis of predictors for aromatase inhibitor-associated rapid bone loss.

Variable	Beta	SE	OR (95% CI)	P
Intercept	-6.97	—	—	—
BMI (kg/m^2^)	0.08	0.05	1.09 (0.99-1.19)	0.074
High PLR (0=Low, 1=High >=214)	0.76	0.39	2.14 (1.00-4.56)	0.050
Follow-up Interval (Years)	-1.02	0.37	0.36 (0.17-0.75)	0.006
Baseline Total Hip BMD (g/cm2)	2.44	1.17	11.53 (1.17-113.67)	0.036
RBC count (×10¹²/L)	0.92	0.35	2.50 (1.26-4.95)	0.008
History of Diabetes (0=No, 1=Yes)	-2.12	0.67	0.12 (0.03-0.45)	0.002
AI exposure before baseline DXA (Months)	-0.03	0.02	0.97 (0.94-1.00)	0.045

Beta, Standard regression coefficient; SE, standard error; OR, odds ratio; CI, confidence interval; BMI, body mass index; BMD, bone mineral density; PLR, platelet-to-lymphocyte ratio; AI, aromatase inhibitor; RBC, Red blood cell.

To further validate the stability and clinical utility of the final model, two robustness analyses were performed (**Supplementary Table 3**). First, given the relatively wide confidence interval observed for baseline total hip BMD (OR = 11.53, 95% CI: 1.17–113.67), Firth’s penalized logistic regression and bootstrap resampling (1,000 iterations) were applied. Both methods yielded consistent, stable estimates (Firth’s OR = 10.38, 95% CI: 1.13–102.40, P = 0.038; bootstrap-corrected 95% CI for the coefficient, 0.310–4.757, excluding zero), confirming that baseline hip BMD is a consistent predictor despite its wide standard confidence interval (**Supplementary Table 3**, Section A).

Second, to assess the individual contributions of BMI and diabetes—two variables with marginal significance or limited event numbers—two reduced 6-predictor models were compared against the full model (AUC = 0.735, 95% CI: 0.672–0.798; sensitivity 71.2%, specificity 65.4%). Excluding BMI reduced sensitivity to 61.3% (specificity 73.5%; AUC = 0.729, 95% CI: 0.666–0.791), and excluding diabetes reduced sensitivity to 55.0% (specificity 74.3%; AUC = 0.696, 95% CI: 0.631–0.761). While these exploratory analyses show that both exclusions compromised model sensitivity—a critical property for a clinical screening tool—these findings should be interpreted with caution. Rather than providing definitive evidence for predictor retention, they simply suggest that incorporating BMI and diabetes may help optimize the potential screening utility of the model. (**Supplementary Table 3**, Section B).

**Figure 4:**
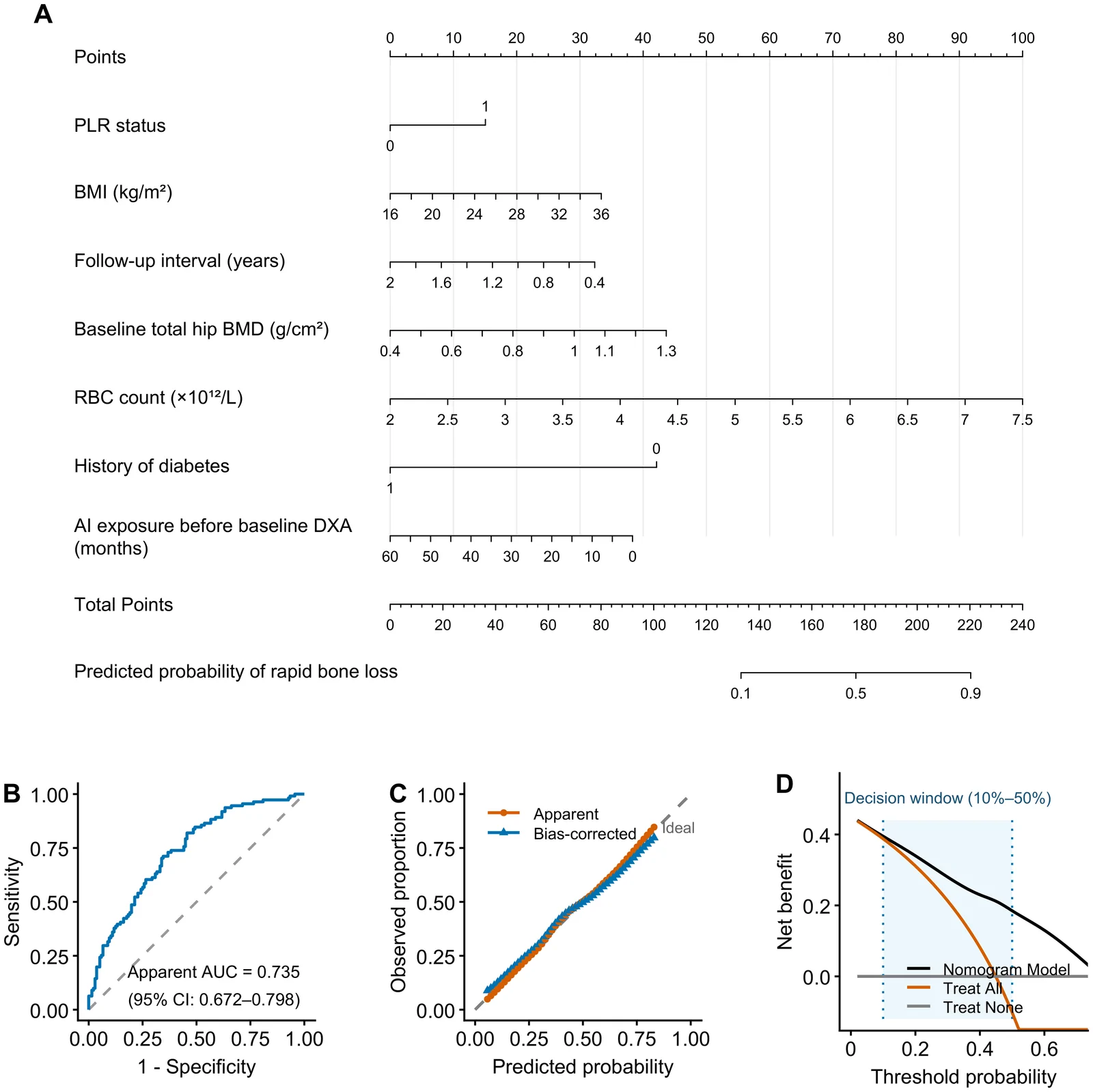
Predictive nomogram and comprehensive performance evaluation. **(A)** Clinical prediction nomogram based on the final seven predictors. **(B)** Apparent ROC curve showing an AUC of 0.735 (95% CI: 0.672–0.798) calculated via the DeLong method. **(C)** Bootstrap calibration plot (1,000 resamples) reporting the optimism-corrected AUC (0.709), calibration slope (0.835), and Brier score (0.219). **(D)** Decision Curve Analysis (DCA) demonstrating positive clinical net benefits across a 10%–50% threshold probability (Pt) decision window. AUC, area under the ROC curve; DCA, decision curve analysis; ROC, receiver operating characteristic.

Based on the results of the multivariable analysis, a graphical nomogram was developed to estimate the risk of AI-associated rapid bone loss (**Figure 4A**). In this model, each predictor is allocated a corresponding point value according to its relative contribution. By summing the points for all variables and projecting the total onto the overall score axis, the individualized probability of rapid bone loss can be readily determined.

The final multivariable logistic regression equation was formulated as follows:


Logit(P)=−6.972+(0.084×BMI)+(0.759×PLR_high)−(1.019×Interval_Years)+(2.445×Hip_BMD_Base)+(0.916×RBC)−(2.123×Diabetes)−(0.032×AI_Duration)

where: BMI is in kg/m^2^, RBC is in ×10^12/^L, Hip_BMD_Base is in g/cm^2^, and AI_Duration is in months; PLR_high is coded as 1 for High [≥214] and 0 for Low; Diabetes is coded as 1 for Yes and 0 for No; and Interval_Years represents the follow-up interval in continuous years and P represents the predicted probability of rapid bone loss. Based on this mathematical framework, we established a simplified clinical point-scoring card to convert these continuous and categorical parameters into integers, allowing for easy bedside application. The complete scoring rules and point-to-probability conversion scale are detailed in **Supplementary Table 6**.

## Validation of the prediction model

### Discrimination

The discriminative performance of the nomogram was assessed using the ROC curve analysis. As depicted in **Figure 4B**, the model exhibited acceptable predictive accuracy, with an AUC of 0.735. The optimal threshold, identified based on the Youden index, was 0.454.

At this cutoff, the model achieved an overall accuracy of 68%, with a sensitivity of 71.2% and a specificity of 65.4%. The positive predictive value (PPV) was 62.7%, while the negative predictive value (NPV) reached 73.6%. These results indicate that the nomogram provides a reasonable capacity to distinguish patients at elevated risk of rapid bone loss from those at lower risk.

### Calibration

Calibration of the model was evaluated using calibration curves in conjunction with the Hosmer–Lemeshow (H–L) goodness-of-fit test, alongside comprehensive bootstrap bias-corrected validation metrics (**Figure 4C**). The calibration plot, derived from bootstrap resampling with 1,000 iterations, showed close agreement between the probabilities estimated by the nomogram and the corresponding observed outcomes. This acceptable calibration was further corroborated by the H–L test, which yielded a non-significant result (χ2 = 4.26, df = 8, P = 0.833), indicating no evidence of a meaningful difference between predicted and actual event rates.

Beyond the H–L test, the model demonstrated moderate prediction accuracy and parameter stability across 1,000 bootstrap resampling iterations, with the probability density distributions of the bootstrapped AUCs and calibration slopes visualized in **Supplementary Figure 4**. The model yielded a bias-corrected Brier score of 0.219 (apparent Brier score: 0.206), a bias-corrected calibration slope of 0.835 (apparent slope: 1.000), and a calibration intercept of -0.013 (**Supplementary Table 7**). Furthermore, after correcting for an overfitting optimism bias of 0.026, the apparent model AUC of 0.735 was adjusted to an optimism-corrected AUC of 0.709, confirming that our predictive framework maintains modest discriminative power. Additionally, to evaluate the model’s calibration stability across different clinical strata, independent calibration curves were constructed for the low-risk (predicted probability <45.4%, n=126) and high-risk (predicted probability ≥45.4%, n=121) subgroups, confirming acceptable predictive accuracy and calibration across both clinical risk strata (**Supplementary Figure 5**).

### Clinical utility

The DCA was conducted to assess the potential clinical utility of the nomogram (**Figure 4D**; **Supplementary Figure 6**). The decision curve for our model (black line) remained consistently above both the ‘treat-all’ strategy (red line) and ‘treat-none’ strategy (gray line) across a wide, clinically meaningful threshold probability range of 10% to 50%. This range represents the critical ‘gray zone’ where clinical decision-making for initiating prophylactic bone-protective therapy is most challenging. Within this range, using the nomogram to guide intervention consistently yielded potential positive net benefits.

Specifically, at a conservative 30% risk threshold, the net benefit of the nomogram was 0.280 (compared to 0.213 for ‘treat-all’). At the optimal clinical cutoff of 45.4%, using the nomogram yielded a net benefit of 0.210, outperforming ‘treat-all’ (0.038), suggesting that model-guided stratification would potentially reduce unnecessary treatment in low-risk patients. At a 50% threshold, using the nomogram yielded a potential net benefit of 0.185, whereas the ‘treat-all’ strategy resulted in a net clinical harm of -0.102. These quantified potential net benefit values suggest the potential clinical utility of our nomogram, pending prospective validation. It should be noted that decision curve analysis evaluates hypothetical threshold-based net benefit; it does not demonstrate that use of the model would actually prevent unnecessary treatment, fractures, or clinically meaningful bone loss.

### Sensitivity analysis and site-specific models

To confirm model robustness, we refitted our 7-predictor model across alternative ABLR cutoffs (>2.5%, >3.0%, and >5.0%), demonstrating stable performance (**Supplementary Table 5**; **Supplementary Figure 2**). Furthermore, to address potential misclassification from the composite ‘worst-site’ approach, independent site-specific models were validated for the lumbar spine and total hip separately. As shown in **Supplementary Figure 3**, our model maintained comparable discrimination at both individual anatomical sites (lumbar spine model AUC = 0.737; total hip model AUC = 0.731).

To evaluate potential mathematical coupling and the influence of pre-baseline AI exposure, we performed further sensitivity analyses by excluding the follow-up interval, the AI exposure before baseline DXA, or both variables from the predictor set (**Supplementary Table 4**). Even after excluding the follow-up interval (Model 2), the model maintained stable and acceptable discrimination (AUC = 0.707, 95% CI: 0.643–0.771), indicating that its predictive power is robustly driven by biological markers rather than mathematical coupling. Similarly, excluding the AI duration prior to baseline DXA (Model 3) or both variables (Model 4) yielded stable predictive capabilities, with AUCs of 0.725 (95% CI: 0.663–0.788) and 0.697 (95% CI: 0.632–0.763), respectively. All models showed adequate goodness-of-fit, with Hosmer-Lemeshow test P-values above 0.05.

### Machine learning comparison

**Figure 5:**
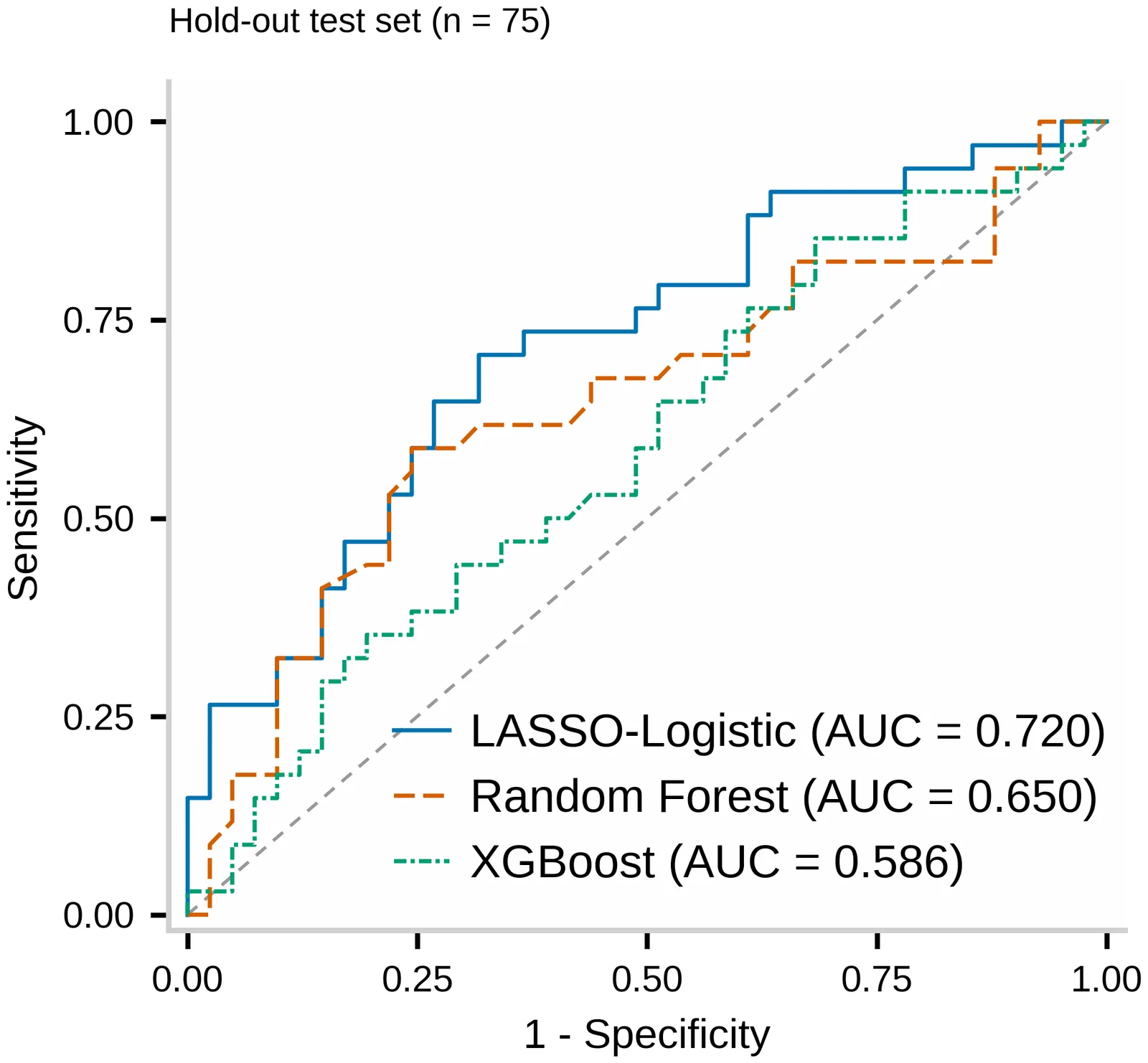
Exploratory model comparisons on the independent test set. Overlaid ROC curves comparing LASSO-logistic regression (Test AUC = 0.720), Random Forest (Test AUC = 0.650), and XGBoost (Test AUC = 0.586) on the independent 30% test partition (n=75, trained on n=172).

Beyond standard internal validation, we performed an exploratory benchmarking comparison against non-linear machine-learning algorithms. To avoid data leakage, the cohort was first split 7:3 into training and test sets; LASSO feature selection was performed using the training set only, and the resulting seven variables were used to train the logistic regression, Random Forest, and XGBoost models, each evaluated once on the untouched test set (n = 75). On the hold-out set, the LASSO-logistic model achieved a Test AUC of 0.720, compared with 0.650 for Random Forest and 0.586 for XGBoost (**Figure 5**; complete hyperparameter tuning grids and validation metrics are detailed in **Supplementary Table 8**). Bootstrap-based optimism-corrected internal validation of the logistic model further supported its stability, yielding an optimism-corrected AUC of 0.709 (apparent AUC = 0.735), a calibration slope of 0.835 (apparent slope = 1.000), and a Brier score of 0.219 (**Figure 4B**; **Supplementary Table 7**). Given the modest size of the hold-out set, this comparison should be interpreted as exploratory rather than a definitive demonstration of superiority; the lower performance of the non-linear models likely reflects overfitting under limited training data rather than an inherent advantage of linear modeling. These findings nonetheless support the parsimonious LASSO-logistic model as a stable and clinically practical choice for the present dataset.

## Discussion

In this ten-year real-world investigation, we developed and internally validated a novel LASSO-based prediction model to estimate the risk of rapid bone loss associated with AI therapy in patients with hormone receptor-positive (HR+) breast cancer. To our knowledge, existing studies have largely concentrated on osteoporosis (a static endpoint) or fracture events as the primary outcomes (6, 11). In contrast, our study redirected the focus from static BMD measurements to the dynamic rate of bone loss, defined as an ABLR>4% (9). This strategy more effectively reflects the progression of bone loss associated with AI therapy. The final model incorporated seven easily accessible clinical variables and demonstrated moderate discrimination (AUC = 0.735) and calibration (H–L test P = 0.833). Furthermore, our sensitivity analyses demonstrated that the model’s discriminative ability and the significance of key predictors remained relatively stable across various ABLR cutoffs (ranging from 2.5% to 5.0%), suggesting that the model’s performance is broadly consistent across these thresholds and supporting > 4.0% as a reasonable, balanced cutoff for exploratory screening. Ultimately, our findings suggest that this prediction model may serve as a promising, exploratory screening tool to assist breast surgeons and oncologists in risk-stratifying patients early, although future prospective multicenter validation remains a prerequisite before formal clinical application.

We adopted a site-specific approach (spine or hip) to define rapid bone loss. Since trabecular bone (spine) is metabolically more active and sensitive to estrogen deprivation than cortical bone (hip), discordance in bone loss rates between sites is common (12). Defining the outcome based on the site with the most severe loss ensures that patients with site-specific rapid deterioration are not overlooked.

Aromatase inhibitor-associated bone loss (AIBL) is characterized by a rapid acceleration of bone turnover, often exceeding the physiological loss rate of postmenopausal women by two- to four-fold (4). Traditional guidelines recommend intervention primarily when the T-score falls below -2.0 or -2.5 (7). However, this “treat-to-target” strategy may be too reactive. Patients with a rapid loss rate (e.g., ABLR> 4%) may experience microarchitectural deterioration and an increased risk of fracture even before their BMD drops into the osteoporotic range (13). Our nomogram fills this gap by identifying these “rapid losers” at the initiation or early stages of AI therapy. DCA suggested a potential net benefit of model-guided risk stratification over the treat-all or treat-none strategies across the 10%–50% threshold range; whether this translates into improved clinical outcomes requires prospective evaluation.

The variables identified through LASSO selection provide biologically plausible insights. Notably, a history of diabetes mellitus was associated with a lower risk of rapid bone loss (OR = 0.12). However, this estimate should be interpreted with caution, as only 3 diabetic patients were present in the rapid bone loss group, and the wide confidence interval reflects this limited number of events. Although sensitivity analysis indicated that excluding diabetes reduced model performance, this finding should not be over-interpreted as a stable or generalizable protective effect. This observation is broadly consistent with prior reports that BMD is often relatively preserved in type 2 diabetes, but must be considered within the context of the well-recognized “diabetes-bone paradox,” in which preserved BMD coexists with increased fragility fracture risk due to impaired bone quality (e.g., via advanced glycation end-product accumulation and increased bone marrow adiposity) (14, 15). Accordingly, the “protective” effect observed here pertains specifically to BMD preservation, should not be interpreted as indicating a lower risk of fragility fractures, and should not inform clinical decisions regarding diabetes management without confirmation in larger cohorts.

In addition, a longer follow-up duration was associated with a lower ABLR. This finding is consistent with the natural course of the AIBL, in which the most pronounced decline typically occurs within the first 1–2 years of treatment, followed by a subsequent plateau (9). This observation highlights the critical importance of close and proactive monitoring during the first year of AI therapy.

Our findings suggest a potential association between elevated PLR and rapid bone loss (OR = 2.14, 95% CI: 1.00–4.56, P = 0.050); however, given that this result was of only borderline statistical significance—and that the association was no longer significant when PLR was modeled as a continuous variable (OR = 1.002, 95% CI: 0.999–1.004, P = 0.249)—this finding should be regarded as preliminary rather than conclusive. With this caveat, the observation may offer indirect, hypothesis-generating support for the concept of “osteoimmunology” (16). As a widely used indicator of systemic inflammation, PLR reflects a critical imbalance between innate immune components (platelets) and adaptive immune responses (lymphocytes). The association between elevated PLR and accelerated bone loss is likely driven by multiple interacting mechanisms (17). Specifically, platelets actively drive bone resorption by releasing extracellular vesicles and soluble mediators that disrupt the RANKL/OPG balance, creating a strongly pro-osteoclastogenic microenvironment (18). Concurrently, AI-induced estrogen deprivation triggers chronic low-grade inflammation, activating immune cells to secrete cytokines (e.g., TNF-α, IL-6) that further stimulate osteoclast activity and induce osteoblast apoptosis (19). Therefore, a high PLR—reflecting relative thrombocytosis and lymphopenia—may plausibly parallel a systemic pro-inflammatory, pro-osteolytic state; however, this mechanistic interpretation remains speculative in the context of the present findings and requires confirmation in studies with direct measurement of relevant biomarkers.

Another unexpected finding was the baseline-dependent pattern of bone loss, whereby patients with higher initial BMD showed a statistically significant association with rapid decline. Two non-biological explanations should also be considered. First, regression to the mean may contribute: baseline measurements partly elevated by random variability tend to be lower at follow-up, manifesting as a greater apparent decline. Second, because the denominator of the ABLR is baseline BMD, baseline BMD enters both sides of the association, and this mathematical coupling can itself generate a positive correlation between baseline BMD and percentage loss. Importantly, because of our retrospective observational design, this pattern must be interpreted with caution as a statistical correlation rather than a direct causal relationship. Current clinical protocols generally recommend routine BMD surveillance only after T-scores fall below defined osteopenic or osteoporotic thresholds. As a result, women with normal baseline bone mass are often categorized as low-risk, leading to less intensive monitoring and infrequent use of prophylactic bone-modifying agents. However, our results suggest that this subgroup actually possesses the greatest bone reserve to lose and may experience the most pronounced reduction following abrupt AI-induced estrogen deprivation. Accordingly, these patients with initially normal BMD should undergo cautious, standard clinical monitoring. If confirmed in prospective cohorts, these findings may highlight the need to direct greater clinical vigilance toward this under-recognized population to mitigate early microarchitectural deterioration.

Calcium and vitamin D supplementation were included as covariates in the analysis; however, they did not demonstrate a statistically significant protective effect against rapid bone loss (20). This finding suggests that routine calcium and vitamin D supplementation alone may be inadequate to offset the pronounced bone-resorptive effects of aromatase inhibitors. For patients at elevated risk, more potent antiresorptive therapies—such as zoledronic acid or denosumab—may be required to achieve effective skeletal protection (21). However, the lack of statistical significance may also be attributable to the relatively small sample size, and thus warrants further validation in larger cohorts.

RBC count also emerged as an independent predictor of rapid bone loss in our model; however, the underlying biological mechanism remains unclear and this association should be interpreted with caution. Prior studies have reported positive associations between RBC count and BMD in postmenopausal women (22), and BMD loss itself has been linked to declining blood cell counts in longitudinal cohorts (23), consistent with a shared bone marrow microenvironment in which hematopoiesis and osteogenesis may be co-regulated, potentially via bone marrow adipose tissue (24). Nonetheless, given the exploratory nature of this analysis, we regard RBC count as a hypothesis-generating finding rather than a validated biological marker, and it should not be used to guide clinical decision-making at this stage.

Several limitations should be acknowledged. First, the retrospective, single-center design introduces potential selection bias and precludes assessment of medication adherence; moreover, the cohort consisted entirely of Asian Chinese women from a single geographic site, which—together with the modest sample size—precludes external validation and limits generalizability. Second, the ten-year enrollment window (2016–2025), despite locally uniform AI regimens and densitometry hardware, cannot exclude hidden confounding from gradual shifts in diet, ancillary bone-protective care, or testing conditions. Third, bone-turnover biomarkers and clinical fracture outcomes were not routinely available for all participants. Fourth, because the baseline DXA scan was performed after AI treatment had already started (median pre-baseline exposure: 4.0 months), an unknown proportion of early therapy-induced bone loss may have occurred before study entry; consequently, the model predicts rapid bone loss occurring after the first available DXA measurement rather than from the true initiation of therapy, and covariate adjustment for pre-baseline exposure cannot fully compensate for this. Fifth, the PLR (214), NLR (2.4), and LDL-C (2.8 mmol/L) cutoffs were derived from the same dataset using the Youden index, introducing optimism bias that bootstrap validation can only partially mitigate; these cutoffs require independent external validation before clinical use. Finally, although bootstrap-based internal validation and machine-learning benchmarking supported the model’s stability, all findings should be interpreted within these constraints.

Ultimately, this investigation developed a preliminary, internally validated prediction model, using routinely available clinical and laboratory variables, for identifying patients potentially at elevated risk of accelerated bone loss during AI therapy. The model demonstrated modest optimism-corrected discrimination (AUC 0.709) and acceptable bias-corrected calibration (slope 0.835) within this single-center cohort, suggesting that such an approach could, in principle, support risk stratification using data already collected in routine clinical practice. However, given the absence of external validation and the modest sample size, these findings should be regarded as hypothesis-generating rather than clinically actionable at this stage. Prospective, multicenter validation is needed before this model, or similar approaches, could be considered to guide individualized bone-preserving strategies in clinical practice.

## Data Availability

The raw data supporting the conclusions of this article will be made available by the authors, without undue reservation.
