# Supplementary material for: Construction and internal validation of a risk prediction model for rapid bone loss during aromatase inhibitor therapy
Source: Front Oncol. 2026 Aug 26;16:1854781. doi: 10.3389/fonc.2026.1854781 (PMC13553356; doi:10.3389/fonc.2026.1854781)
Supplement: Supplementary file 1 [file Table1.docx]

**Supplementary Table 1.  Overview of candidate variables, clinical definitions, coding methods, and missing rates in the final analyzed cohort (N=247).**

| **Variable Name** | **Clinical Definition** | **Unit** | **Variable Type & Coding Method** | **Missing Rate (%)** |
| --- | --- | --- | --- | --- |
| **Age** | Patient age at study entry | Years | Continuous | 0% |
| **BMI** | Body mass index | kg/m2 | Continuous | 0% |
| **Meno_Duration** | Time elapsed since menopause | Years | Categorical:  0 = ≤5 years;  1 = >5 years | 0% |
| **History_Fracture** | Prior history of fragility fractures | — | Binary: 0 = No; 1 = Yes | 0% |
| **Hypertension** | History of diagnosed hypertension | — | Binary: 0 = No; 1 = Yes | 0% |
| **Diabetes** | History of diagnosed diabetes mellitus | — | Binary: 0 = No; 1 = Yes | 0% |
| **Glucocorticoids** | History of prior glucocorticoid use | — | Binary: 0 = No; 1 = Yes | 0% |
| **Ki-67** | Ki-67 proliferation index | % | Categorical: 0 =Low(<20%); 1 = High (≥  20%) | 0% |
| **Hip_BMD_Base** | Bone mineral density of total hip at study entry | g/cm2 | Continuous | 0% |
| **LS_BMD_Base** | Bone mineral density of lumbar spine (L1-L4) at study entry | g/cm2 | Continuous | 0% |
| **Interval_Months** | Time elapsed between baseline and follow-up scans | Years | Continuous | 0% |
| **Clin_Stage_Cat** | Tumor clinical stage at diagnosis | — | Categorical: 0 = Stage I; 1 = Stage II-IV | 0% |
| **T_Stage_Cat** | Tumor size staging | — | Categorical: 0 = Stage T1; 1 = Stage T2-T4 | 0% |
| **AI_Type** | Type of aromatase inhibitor received | — | Categorical: Letrozole, Anastrozole, or Exemestane | 0% |
| **Ca_VitD_Supp** | Adherence to Calcium and Vitamin D supplements | — | Binary: 0 = No; 1 = Yes | 0% |
| **AI_Duration** | Time elapsed between AI initiation and baseline DXA | Months | Continuous | 0% |
| **Radiotherapy** | Prior history of radiotherapy | — | Binary: 0 = No; 1 = Yes | 0% |
| **Chemotherapy** | Prior history of chemotherapy | — | Binary: 0 = No; 1 = Yes | 0% |
| **Targeted_Therapy** | Prior history of targeted therapy | — | Binary: 0 = No; 1 = Yes | 0% |
| **NLR** | Neutrophil-to-lymphocyte ratio | — | Categorical: 0 = Low (<2.4); 1 = High (≥2.4) | 0% |
| **PLR** | Platelet-to-lymphocyte ratio | — | Categorical: 0 = Low (<214); 1 = High (≥214) | 0% |
| **RBC** | Red blood cell count | ×10^12^/L | Continuous | 0% |
| **WBC** | White blood cell count | ×10^9^/L | Continuous | 0% |
| **NEUT** | Neutrophil count | ×109/L | Continuous | 0% |
| **LYMPH** | Lymphocyte count | ×109/L | Continuous | 0% |
| **Hb** | Hemoglobin level | g/L | Continuous | 0% |
| **PLT** | Platelet count | ×109/L | Continuous | 0% |
| **Cr** | Serum creatinine level | μmol/L | Continuous | 4% |
| **TBil** | Total bilirubin | μmol/L | Continuous | 0% |
| **TP** | Total protein | g/L | Continuous | 0% |
| **ALT** | Alanine aminotransferase | U/L | Continuous | 0% |
| **AST** | Aspartate aminotransferase | U/L | Continuous | 0% |
| **ALP_Cat** | Alkaline phosphatase | U/L | Categorical: 0 = Normal (≤100); 1 = High (>100) | 0% |
| **LDL_Cat** | Low-density lipoprotein cholesterol | mmol/L | Categorical:  0 = Optimal (≤2.8);  1 = Elevated (>2.8) | 0% |
| **CA** | Serum calcium levels | mmol/L | Continuous | 0% |
| **P** | Serum phosphorus levels | mmol/L | Continuous | 0% |
| **Ca_P_Product** | Calcium-phosphorus product | mmol^2^/L^2^ | Continuous | 0% |
| **Ca_P_Ratio** | Calcium-phosphorus ratio | — | Continuous | 0% |
| **New_Fracture** | Incident fragility fractures during follow-up | — | Binary: 0 = No; 1 = Yes | 0% |
| **VitD** | 25-hydroxyvitamin D [25(OH)D] level | ng/mL | Continuous | 70% |
| **PRL** | Prolactin level | μg/L | Continuous | 64% |
| **E2** | Estradiol level | μ*mol*/*L* | Continuous | 64% |

*Abbreviations: ABLR, annual bone mineral density loss rate; AI, aromatase inhibitor; BMI, body mass index; BMD, bone mineral density; DXA, dual-energy X-ray absorptiometry; NLR, neutrophil-to-lymphocyte ratio; PLR, platelet-to-lymphocyte ratio; RBC, red blood cell; WBC, white blood cell; PRL, prolactin; E2, Estradiol.*
