# Supplementary material for: Construction and internal validation of a risk prediction model for rapid bone loss during aromatase inhibitor therapy
Source: Front Oncol. 2026 Aug 26;16:1854781. doi: 10.3389/fonc.2026.1854781 (PMC13553356; doi:10.3389/fonc.2026.1854781)
Supplement: Supplementary file 2 [file Table2.docx]

**Supplementary Table 2.** Structured overview of screening and exclusion criteria applied during patient selection (2016–2025)

| **exclude the cause** | **number** | **Proportion of the total** |
| --- | --- | --- |
| No DXA scan data | 221 | 0.42 |
| Only one DXA scan performed | 130 | 0.25 |
| Bone Metastasis at baseline | 28 | 0.05 |
| Use of bone-modifying agents before baseline or during follow-up | 52 | 0.10 |
| Follow-up interval not meeting criteria | 45 | 0.09 |
| Short duration of AI therapy(<6 months) | 39 | 0.07 |
| Missing key clinical data | 10 | 0.02 |
|  |  |  |
|  |  |  |
