# Supplementary material for: Construction and internal validation of a risk prediction model for rapid bone loss during aromatase inhibitor therapy
Source: Front Oncol. 2026 Aug 26;16:1854781. doi: 10.3389/fonc.2026.1854781 (PMC13553356; doi:10.3389/fonc.2026.1854781)
Supplement: Supplementary file 3 [file Table3.docx]

**Supplementary Table 3.** **Supplementary Table 4. Model stability and robustness validation**

**Section A. Comparison of standard, penalized (Firth's), and bootstrap-corrected estimates**

| **Predictor** | **Standard Logistic Regression**  **β (SE)** | **Standard OR (95% CI)** | **Standard**  **P-value** | **Firth's Penalized Regression**  **β(SE)** | **Penalized OR (95% CI)** | **Penalized**  **P-value** | **Bootstrap-Corrected**  **β (95% CI)** |
| --- | --- | --- | --- | --- | --- | --- | --- |
| **Intercept** | -6.972 (1.988) | — | <0.001 | -6.637 (1.919) | — | <0.001 | -6.972 (-12.111 to -3.042) |
| **BMI** | 0.084 (0.047) | 1.09 (0.99–1.19) | 0.074 | 0.081 (0.046) | 1.08 (0.99–1.19) | 0.075 | 0.084 (-0.014 to 0.185) |
| **PLR_high** | 0.759 (0.387) | 2.14 (1.00–4.56) | 0.050 | 0.731 (0.376) | 2.08 (1.00–4.43) | 0.051 | 0.759 (0.038 to 1.621) |
| **Interval_Months** | -1.019 (0.374) | 0.36 (0.17–0.75) | 0.006 | -0.979 (0.364) | 0.38 (0.18–0.76) | 0.006 | -1.019 (-1.858 to -0.275) |
| **Hip_BMD_Base** | 2.445 (1.168) | 11.53 (1.17–113.67) | 0.036 | 2.340 (1.134) | 10.38 (1.13–102.40) | 0.038 | 2.445 (0.310 to 4.757) |
| **RBC** | 0.916 (0.348) | 2.50 (1.26–4.95) | 0.008 | 0.865 (0.334) | 2.37 (1.26–4.81) | 0.006 | 0.916 (0.196 to 1.856) |
| **Diabetes** | -2.123 (0.673) | 0.12 (0.03–0.45) | 0.002 | -1.943 (0.619) | 0.14 (0.04–0.44) | <0.001 | -2.123 (-17.747 to -0.982) |
| **AI_Duration** | -0.032 (0.016) | 0.97 (0.94–1.00) | 0.045 | -0.030 (0.015) | 0.97 (0.94–1.00) | 0.042 | -0.032 (-0.061 to -0.007) |

**Note: Bootstrap estimates and their 95% confidence intervals (CI) were generated using 1,000 resampling repetitions. Under Firth's penalized likelihood and Bootstrap resampling, the parameter estimate for baseline total hip BMD was successfully stabilized and narrowed, confirming the robustness and statistical stability of the clinical prediction model.*

**Section B. Full model vs. BMI-excluded model performance**

| **Model** | **Predictors** | **AUC (95% CI)** | **Sensitivity** | **Specificity** | **PPV** | **NPV** |
| --- | --- | --- | --- | --- | --- | --- |
| Full model | 7 | 0.735 (0.674–0.797) | 71.2% | 65.4% | 62.7% | 73.6% |
| Excl. BMI | 6 | 0.729 (0.666–0.791) | 61.3% | 73.5% | 65.4% | 69.9% |
| Excl. Diabetes | 6 | 0.696 (0.631–0.761) | 55.0% | 74.3% | 63.5% | 66.9% |
