# Supplementary material for: Construction and internal validation of a risk prediction model for rapid bone loss during aromatase inhibitor therapy
Source: Front Oncol. 2026 Aug 26;16:1854781. doi: 10.3389/fonc.2026.1854781 (PMC13553356; doi:10.3389/fonc.2026.1854781)
Supplement: Supplementary file 4 [file Table4.docx]

**Supplementary Table 4.** Sensitivity analysis of model performance excluding the follow-up interval and AI exposure before baseline DXA.

| **Model Scenario** | **Predictors Included** | **AUC (95% CI)** | **Sensitivity (%)** | **Specificity (%)** | **PPV (%)** | **NPV (%)** | **H-L Test (P-value)** |
| --- | --- | --- | --- | --- | --- | --- | --- |
| Model 1: Full Model | All 7 predictors (BMI retained despite marginal P-value) | 0.735 (0.674–0.797) | 71.2 | 65.4 | 62.7 | 73.6 | 0.883 |
| Model 2: Excluding Follow-up Interval | BMI, High PLR, Baseline Hip BMD, RBC count, Diabetes, AI exposure before baseline DXA | 0.707 (0.643–0.771) | 66.7 | 65.4 | 61.2 | 70.6 | 0.724 |
| Model 3: Excluding AI exposure before baseline DXA | BMI, High PLR, Follow-up Interval, Baseline Hip BMD, RBC count, Diabetes | 0.725 (0.663–0.788) | 74.8 | 60.3 | 60.6 | 74.5 | 0.305 |
| Model 4: Excluding Both | BMI, High PLR, Baseline Hip BMD, RBC count, Diabetes | 0.697 (0.632–0.763) | 63.1 | 67.6 | 61.4 | 69.2 | 0.674 |

**Abbreviations:** AUC, area under the receiver operating characteristic curve; CI, confidence interval; H-L, Hosmer–Lemeshow; NPV, negative predictive value; PLR, platelet-to-lymphocyte ratio; PPV, positive predictive value; RBC, red blood cell.
