# Supplementary material for: Construction and internal validation of a risk prediction model for rapid bone loss during aromatase inhibitor therapy
Source: Front Oncol. 2026 Aug 26;16:1854781. doi: 10.3389/fonc.2026.1854781 (PMC13553356; doi:10.3389/fonc.2026.1854781)
Supplement: Supplementary file 5 [file Table5.docx]

**Supplementary Table 5. Predictive performance of the risk model under different annual bone mineral density loss rate (ABLR) cutoffs.**

| **Cutoff Threshold** | **Patients,n (%)** | **AUC (95% CI)** | **Sensitivity (%)** | **Specificity (%)** | **PPV (%)** | **NPV (%)** | **Hosmer-Lemeshow (**  **P-value)** |
| --- | --- | --- | --- | --- | --- | --- | --- |
| **ABLR > 2.5%** | 153  (61.9%) | 0.694 (0.627–0.761) | 57.5 | 73.4 | 77.9 | 51.5 | 0.826 |
| **ABLR > 3.0%** | 138  (55.9%) | 0.706 (0.640–0.771) | 79.0 | 55.0 | 69.0 | 67.4 | 0.817 |
| **ABLR> 4.0%** | 111 (44.9%) | 0.735 (0.672–0.798) | 71.2 | 65.4 | 62.7 | 73.6 | 0.833 |
| **ABLR > 5.0%** | 85  (34.4%) | 0.723 (0.658–0.788) | 75.3 | 62.3 | 51.2 | 82.8 | 0.489 |

*Abbreviations: ABLR, annual bone mineral density loss rate; AUC, area under the receiver operating characteristic curve; CI, confidence interval; LSC, least significant change; NPV, negative predictive value; PPV, positive predictive value.*
