# Supplementary material for: Construction and internal validation of a risk prediction model for rapid bone loss during aromatase inhibitor therapy
Source: Front Oncol. 2026 Aug 26;16:1854781. doi: 10.3389/fonc.2026.1854781 (PMC13553356; doi:10.3389/fonc.2026.1854781)
Supplement: Supplementary file 6 [file Table6.docx]

**Supplementary Table 6. LASSO parameters, full model equation, and exact point-scoring rules for the Nomogram.**

**Part A: LASSO Variable Selection Parameters**

- **Optimal penalty parameter (λ_1se_)**: 0.041 (representing the 1-standard error criterion)
- **Minimum penalty parameter (λ_min_)**: 0.015
- **Variables with non-zero coefficients at λ_1se_**: BMI, PLR_high, Interval_Years, Hip_BMD_Base, RBC, Diabetes, AI_Duration.

**Part B: Full Multivariable Model Equation**

The log-odds of rapid bone loss (RBL) is defined as:
ln($\frac{P}{1-P}$)=−6.972+(0.084×BMI)+(0.759×PLR_high)−(1.019×Interval_Years)+(2.445×Hip_BMD_Base)+(0.916×RBC)−(2.123×Diabetes)−(0.032×AI_Duration)

Where P is the predicted probability of rapid bone loss.

**Part C: Nomogram Point-Scoring Card**

To calculate the total risk score for an individual patient, sum the points assigned to each of the seven predictors according to the exact rules below:

| **Predictor Variable** | **Clinical Value / Category** | **Score Range (pts)** | **Point-Scoring Formula / Rule** |
| --- | --- | --- | --- |
| **RBC Count** | Continuous (×10^12^/L) | **0 to 100.0** | Points=20.8768×(RBC−2.22)  *e.g., RBC = 4.50 → 47.6 points.* |
| **Interval_Years** | Continuous (Years) | **0 to 34.8** | Points=23.2064×(2.00− Interval_Years)  *e.g., Interval = 1.00 year →23.2 points.* |
| **Hip_BMD_Base** | Continuous (g/cm2) | **0 to 44.5** | Points=55.6916×(Hip_BMD_Base−0.45)  *e.g., BMD = 0.85 →22.3 points.* |
| **History of Diabetes** | No (0)  Yes (1) | **48.4** or **0** | *Patients without a history of diabetes are assigned 48.4 points.* |
| **AI_Duration** | Continuous (Months) | **0 to 41.8** | Points=0.7333×(57.00−AI_Duration)  *e.g., Duration = 12.0 months →33.0 points.* |
| **PLR_high** | Low  (0, < 214)  High  (1, ≥214) | **0 or 17.3** | *An elevated platelet-to-lymphocyte ratio adds 17.3 points.* |
| **BMI** | Continuous (kg/m2) | **0 to 21.5** | Points=19162.×(BMI−17.33)  *e.g., BMI = 25.0 →14.7 points.* |

**Part D: Total Points to Probability Conversion Scale**

By summing the points from the seven variables, the **Total Points** (ranging from 0 to 431.5) can be mapped to the predicted probability (P) of rapid bone loss:

| **Total Points** | **Predicted Probability of Rapid Bone Loss (*P*)** |
| --- | --- |
| < 110 | < 3% |
| 130 | ~ 5% |
| 150 | ~ 14% |
| 170 | ~ 29% |
| 187 | ~ 45.4% |
| 200 | ~ 60% |
| 220 | ~ 78% |
| 240 | ~ 90% |
| > 260 | > 95% |

*Abbreviations: ABLR, annual bone mineral density loss rate; BMI, body mass index; BMD, bone mineral density; NLR, neutrophil-to-lymphocyte ratio; PLR, platelet-to-lymphocyte ratio; RBC, red blood cell.*
