# Supplementary material for: Construction and internal validation of a risk prediction model for rapid bone loss during aromatase inhibitor therapy
Source: Front Oncol. 2026 Aug 26;16:1854781. doi: 10.3389/fonc.2026.1854781 (PMC13553356; doi:10.3389/fonc.2026.1854781)
Supplement: Supplementary file 7 [file Table7.docx]

**Supplementary Table 7. Bootstrap validation and optimism-corrected estimates of the risk prediction model (1,000 resamples).**

| **Performance Metric** | **Apparent Estimate** | **Bootstrap Training Mean** | **Bootstrap Testing Mean** | **Optimism (Overfitting Bias)** | **Bias-Corrected (Optimism-Corrected) Estimate** |
| --- | --- | --- | --- | --- | --- |
| **Discrimination** |  |  |  |  |  |
| Area Under the Curve (AUC) | 0.735 | 0.746 | 0.720 | 0.026 | **0.709** |
| **Calibration** |  |  |  |  |  |
| Calibration Slope | 1.000 | 1.035 | 0.835 | 0.165 | **0.835** |
| Calibration Intercept | 0.000 | 0.012 | -0.013 | 0.013 | **-0.013** |
| **Overall Accuracy** |  |  |  |  |  |
| Brier Score | 0.206 | 0.2 | 0.213 | 0.013 | **0.219** |

**Note: Optimism is calculated as the difference between the mean performance on the bootstrap training samples and the bootstrap test samples across 1,000 resampling iterations. A low optimism value indicates that the model is highly stable and does not suffer from significant overfitting or parameter instability.*
