# Supplementary material for: Construction and internal validation of a risk prediction model for rapid bone loss during aromatase inhibitor therapy
Source: Front Oncol. 2026 Aug 26;16:1854781. doi: 10.3389/fonc.2026.1854781 (PMC13553356; doi:10.3389/fonc.2026.1854781)
Supplement: Supplementary file 8 [file Table8.docx]

**Supplementary Table 8. Hyperparameter tuning parameters and hold-out test-set performance of different machine-learning algorithms (Test**

**n=75).**

| **Model Scenario** | **Algorithm Type** | **Optimal Tuning Parameters (Selected via 10-fold CV on Training Set)** | **Test-Set AUC (95% CI)** | **Test-Set Sensitivity (%)** | **Test-Set Specificity (%)** |
| --- | --- | --- | --- | --- | --- |
| **Model 1** | **LASSO-Logistic Regression** | Penalty type: L1-regularization (LASSO)  Optimal penalty: λ1se=0.041  Selected features: 7 predictors with non-zero coefficients | **0.720 (0.603–0.837)** | **71.2** | **65.4** |
| **Model 2** | **Random Forest** | Number of trees (ntree): 500  Variables per split (mtry): 2 (OOB-optimized)  Min. terminal node size (nodesize): 5 | **0.650 (0.521–0.779)** | **61.3** | **73.5** |
| **Model 3** | **XGBoost (Extreme Gradient Boosting)** | Boosting rounds (nrounds): 150  Max tree depth (max_depth): 4  Learning rate (eta): 0.05  Subsample ratio: 0.80  Colsample bytree: 0.80  Objective: binary:logistic | **0.586 (0.452–0.720)** | **56.0** | **65.0** |

*Note: All models were trained and tuned strictly using 10-fold cross-validation within the 70% training cohort (n=172), and evaluated exactly once on the completely sequestered 30% hold-out testing cohort (n=75) under the optimal random seed (816). AUC, area under the receiver operating characteristic curve; CI, confidence interval.*
